# The Society of Chemical Industry and Its Work

**Published:** 1919-07-26

**Authors:** 


### CHEMISTS IN CONFERENCE.

### The Society of Chemical Industry and its work.

Rather less than forty years ago a small band of enthusiastic young men founded in London the Society of Chemical Industry. At that time the chemist in Germany was already given a leading place in national life and organisation; in England his existence, except as a dispenser of medicine, was scarcely recognised by the nation at large, and for many years the Society had an uphill struggle to obtain for itself a position in which it might hope to accomplish its purpose. But success has crowned the effort, the Society has become a vast and powerful organisation, and the industry with which it is concerned is achieving its rightful place in our national life. The Society has a membership of 5,000 representatives of practically all parts of the world, with branches in Glasgow, Edinburgh, Liverpool, Manchester, Bristol, Birmingham, Leeds, and Nottingham, as well as New York, Sydney (N.S.W.), Toronto, and Vancouver. Last week, for the first time since the war, its members met in conference, the proceedings, which extended over three days, taking place at the Mansion House, and the Halls of the Salters', the Clothworkers', and the Goldsmiths' Companies.

On Tuesday morning the Lord Mayor welcomed the members at the Mansion House, and Professor Henry Lewis, D.Sc., President of the Society, gave his presidential address, in the course of which he suggested the possibility of chemists coming to the rescue of a community menaced by coal famine. In the afternoon the subject for consideration was the work of the Inter-Allied Chemical Federation, introduced by Sir William J. Pope, F.R.S., as Chairman of the British Federal Council for Pure and Applied Chemistry, and contributed to, among others, by the eminent French chemist, M. Paul Kestner, President of the Société de Chemie Industrielle. The discussion revealed general agreement on a plan, already well matured, for the co-ordination of international effort in the work of scientific research and investigation, under the direction of an International Research Council. In this scheme British chemists through their Federal Council would take their part in regard to their own particular subject.

On Wednesday the Conference split into two parts for the purpose of giving the whole day to the discussion of two such diverse subjects as "Empire Sugar Production" and "Power Plant in Chemical Works." The members interested in the former subject met at the Clothworkers' Hall, under the chairmanship of the Earl of Denbigh, a zealous advocate of the production of beet-sugar in the United Kingdom. Two years ago the Society of Chemical Industry appointed a Special Committee to inquire into the whole subject of sugar growing in the British Empire, and the Chairman of this Committee, Mr. A. R. Ling, now presented its report. The Committee have collected data from all parts of the Empire on the question whether it is possible to make it self-supporting in the matter of sugar production, and a great deal of interesting information is given in their report. The United Kingdom is the second largest consumer of sugar, with 91 lb. per head per annum, of all the countries in the world, Australia coming first with a consumption of over 1 cwt. per annum. Of the United Kingdom's consumption before the war, 90 per cent. was imported from foreign countries, nine-tenths being beet-sugar mainly grown in Germany and Austria. During the war the world's production has fallen from about 18½ million tons to 17½ million, but that of the British Empire has increased from 3,275,000 tons to 4,394,000, mostly cane-sugar. In the course of the discussion it was suggested that India might largely increase its sugar production, and eventually supply the United Kingdom with the 2½ million tons it now imports from foreign countries.

**Chemists in Conference**—(*continued*).

Much doubt was expressed whether, under any conditions, the production of beet-sugar in this country can successfully compete with cane-sugar in the tropical parts of the Empire. It was generally agreed that there is little or no hope of establishing a beet-sugar industry in this country without considerable assistance in some shape or form from the State. On the whole the Conference created the impression that our wisest policy is to develop fully the cultivation of the sugar-cane in our tropical Dominions.

The Salters' Hall's section of the Conference devoted the whole of Thursday to the subjects of "Dyestuffs, Synthetic Drugs, and Associated Products." In point of fact the re-establishment of the British dye industry and the part to be taken by our chemists in this great task occupied the lion's share of the time. Perhaps this is natural and inevitable in view of the great and immediate importance of the subject. At the present time, however, the topic of synthetic drugs is of only less importance, and it might have been wiser to have given a separate sitting to its consideration.

Thursday morning was occupied by the Goldsmiths' Hall section with "The Chrome Tanning Industry," and the afternoon with "Recent Developments in the Fermentation Industries."

The Conference met for serious business, and very business-like was its tone and procedure. But the social side of such an occasion, bringing together representative men from all parts of the country as well as a sprinkling of distinguished foreign visitors, was not altogether neglected. There was a luncheon at the Connaught Rooms and a dinner at the Savoy Hotel, Lord Moulton, an old friend of the Society, making the principal speech at the latter function. There were also two receptions, one at the Imperial College of Science, South Kensington, and the other at the British Scientific Products Exhibition, Westminster, both occasions happily blending the pleasure of an entertainment and the business of the Conference. The whole programme concluded with an excursion on Friday to Windsor and Taplow.

